# Active carbon sequestration in the Alpine mantle wedge and implications for long-term climate trends

**DOI:** 10.1038/s41598-018-22877-7

**Published:** 2018-03-16

**Authors:** Marco Giovanni Malusà, Maria Luce Frezzotti, Simona Ferrando, Enrico Brandmayr, Fabio Romanelli, Giuliano Francesco Panza

**Affiliations:** 1 Department of Earth and Environmental Sciences, University of Milano-Bicocca, Milano, Italy; 2 Department of Earth Sciences, University of Torino, Torino, Italy; 3 Center for Research Excellence in Science and Technology (CREST), North Carolina Central University, Durham, NC USA; 4 Department of Mathematics and Geosciences, University of Trieste, Trieste, Italy; 5 Institute of Geophysics, China Earthquake Administration, Beijing, People’s Republic of China; 6 Accademia Nazionale dei Lincei, Rome, Italy; 7 ISSO, Arsita, Italy

## Abstract

The long-term carbon budget has major implications for Earth’s climate and biosphere, but the balance between carbon sequestration during subduction, and outgassing by volcanism is still poorly known. Although carbon-rich fluid inclusions and minerals are described in exhumed mantle rocks and xenoliths, compelling geophysical evidence of large-scale carbon storage in the upper mantle is still lacking. Here, we use a geophysical surface-wave seismic tomography model of the mantle wedge above the subducted European slab to document a prominent shear-wave low-velocity anomaly at depths greater than 180 km. We propose that this anomaly is generated by extraction of carbonate-rich melts from the asthenosphere, favoured by the breakdown of slab carbonates and hydrous minerals after cold subduction. The resulting transient network of carbon-rich melts is frozen in the mantle wedge without producing volcanism. Our results provide the first *in-situ* observational evidence of ongoing carbon sequestration in the upper mantle at a plate-tectonic scale. We infer that carbon sequestered during cold subduction may partly counterbalance carbon outgassed from ridges and oceanic islands. However, subducted carbon may be rapidly released during continental rifting, with global effects on long-term climate trends and the habitability of planet Earth.

## Introduction

The long-term carbon budget of planet Earth is largely modulated by the tectonic balance between CO_2_ outgassing to the atmosphere from volcanism, and carbon input to the Earth interior during subduction^1,2^. Carbon input into the mantle depends on the lithologies subducted^3^ and on physical processes, which are only partly understood, that take place in the downgoing slab and in the overlying mantle wedge^4,5^. Some studies suggest that little carbon can be recycled into the mantle^2^, whereas other studies infer that sequestration of subducted carbon in the mantle may be relevant^1^, at least in cooler subduction zones. However, in spite of the potentially relevant implications for climate change and planet habitability, compelling evidence of large-scale carbon storage in the Earth’s upper mantle are still lacking, and it is not clear whether subducted carbon can be effectively sequestered beyond sub-arc depths over geological time scales, or not^1,2,5^.

**Figure 1:**
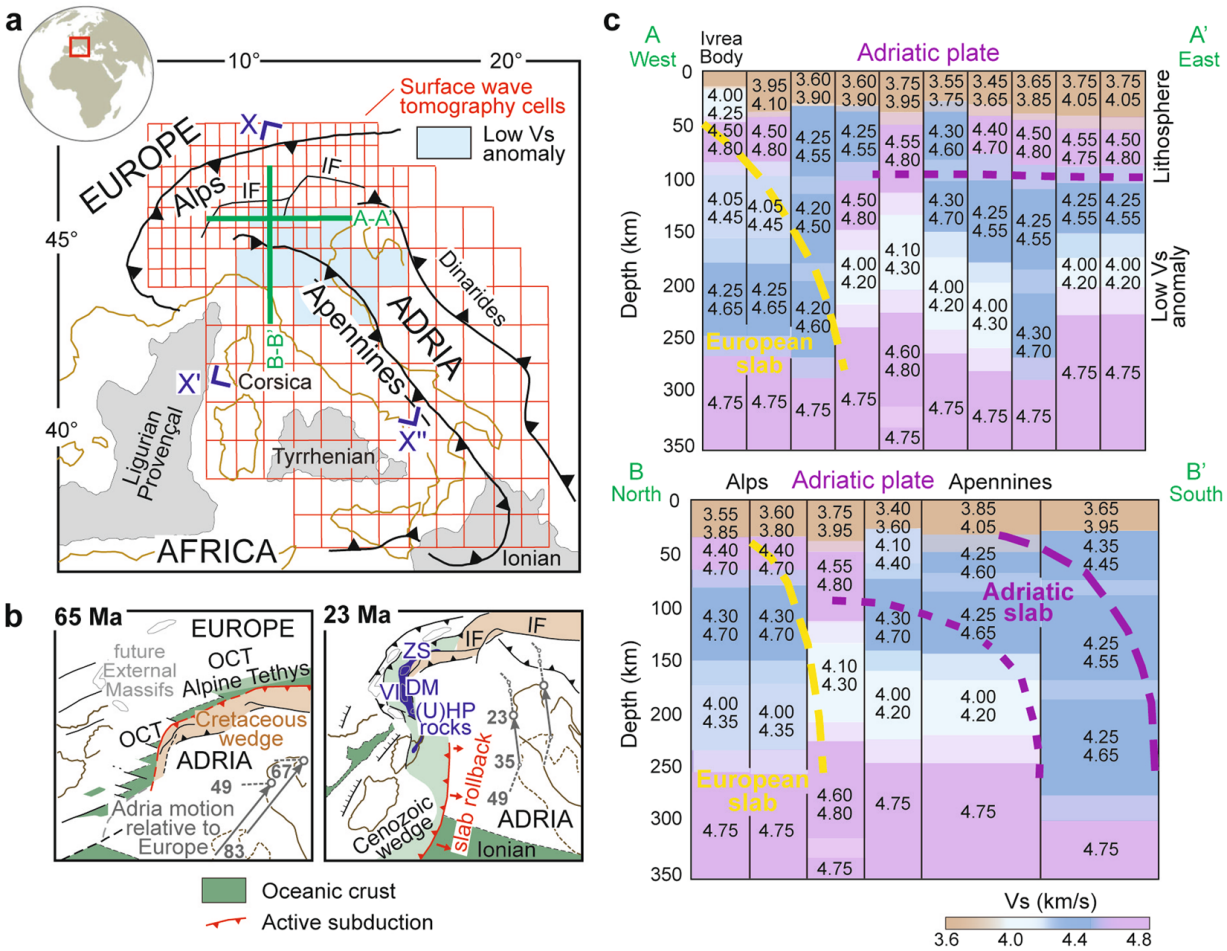
The low Vs anomaly in the Adriatic upper mantle. (**a**) Tectonic sketch map and boundaries of the cellular surface-wave tomography model of the central Mediterranean; cells showing anomalously low Vs values are marked in light blue. (**b**) Cenozoic evolution of Alpine subduction, grey arrows (after ref.^8^) indicate Adria trajectories relative to Europe (numbers = age in Ma); (ultra)high-pressure [(U)HP] units: DM = Dora-Maira, VI = Viso, ZS = Zermatt-Saas; OCT = ocean-continent transition; IF = Insubric Fault. (**c**) Tomographic cross sections showing the low Vs anomaly (light blue) between the European and Adriatic slabs (slab structure after ref.^6^); shaded areas indicate the variability range of layer thickness. Maps and cross sections generated using Inkscape v0.91 (https://inkscape.org).

We combine geodynamic reconstructions of the Adria-Europe plate boundary zone with geophysical imaging and petrological modeling to reveal large-scale carbon processes associated with a complex slab configuration. The geometry of subducted slabs are resolved using recent *P* wave tomography models^6^, and include a SE-dipping European slab to the north and a SW-dipping Adriatic slab to the south (Fig. 1a). Tectonic reconstructions^7,8^ suggest that Alpine subduction was active since the Cretaceous, leading to the consumption of the Alpine Tethys formerly separating the Adriatic and European paleomargins (Fig. 1a,b). Adriatic subduction initially developed south of Corsica, and progressively propagated northward during the Eocene-Oligocene^8^. Since the late Oligocene, the interaction between the SE-dipping European slab and the northward shifting Adriatic slab precluded any major subduction at the Alpine trench^9^. The Adriatic slab began rolling back in the Neogene, leading to the opening of the Ligurian-Provençal and Tyrrhenian backarc basins^10^.

The Alpine subduction wedge mainly consists of (ultra)high-pressure [(U)HP] metamorphic rocks with mineral assemblages that formed during very cold subduction (5–8 °C/km)^8^. Above the remnants of the European slab, body wave tomography models revealed a prominent low-velocity anomaly^6,11,12^ that was tentatively interpreted to result from fluids released by dehydration reactions during the early stages of Alpine subduction^11^. Hypotheses concerning the origin, age and composition of these fluids still remain highly debatable. A reliable analysis of the potential spatial and time relationships between seismic velocity anomalies, slab structure and mineral reactions along the slab interface and in the overlying mantle wedge requires a higher resolution image of the velocity structure of the supra-slab mantle, which is not provided by available body wave tomography models.

## The velocity structure of the supra-slab mantle

**Figure 2:**
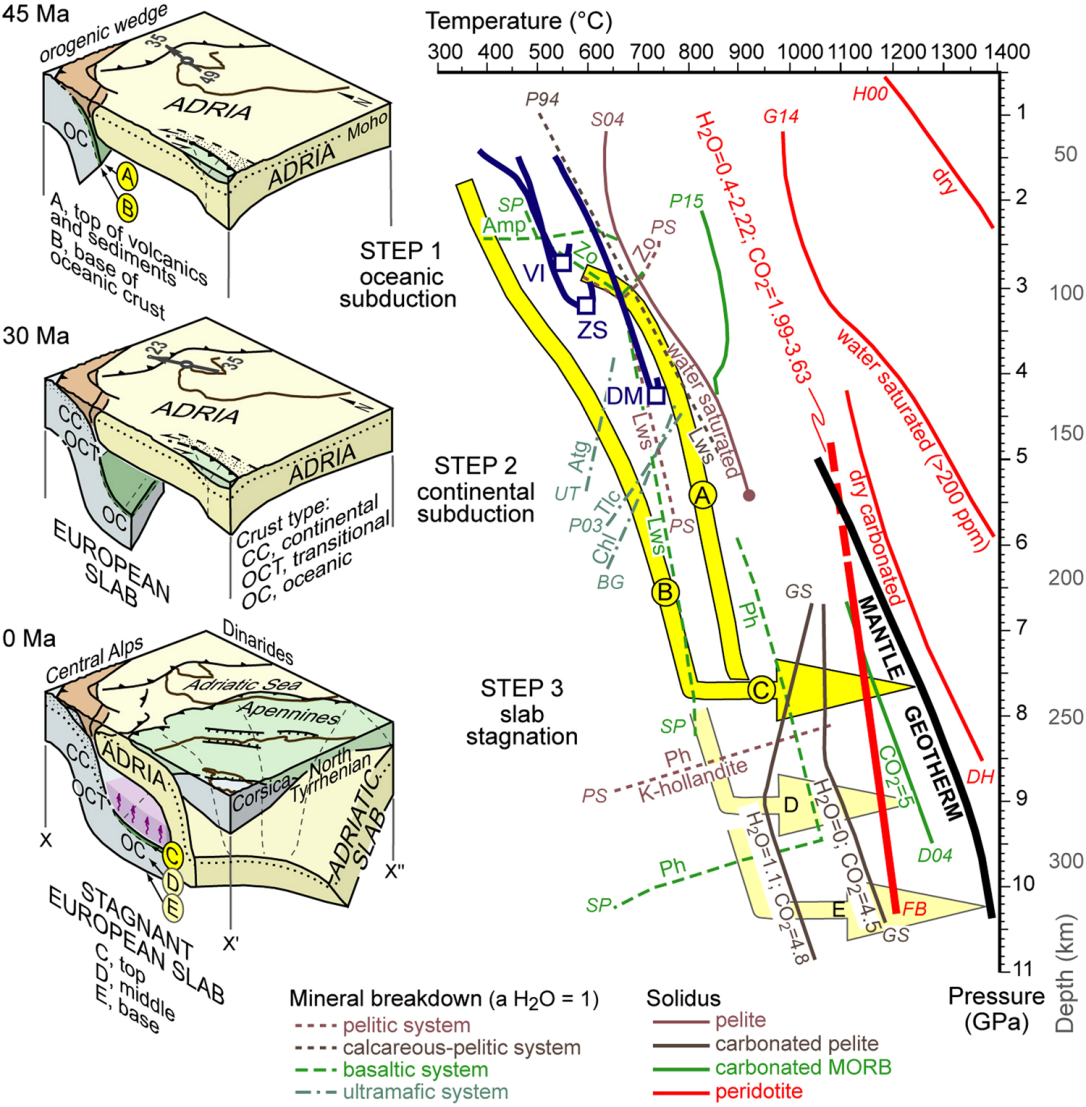
3D reconstruction of Alpine subduction and associated mineral reactions. Prograde pressure-temperature paths of exhumed (U)HP rocks (blue lines)^19–21^ are consistent with modelled pressure-temperature paths of modern subduction zones (yellow arrows A and B, from ref.^25^). Cold subduction favours the preservation of carbonates and hydrous minerals (e.g. phengite) to asthenospheric depths. Progressive increase in slab temperature (yellow arrow C) towards mantle values (black line, from ref.^27^) during slab stagnation induces melting of metasediments (GS^45^, S04^46^) and carbonated metabasics (D04^47^). Consequent generation of carbonate-rich hydrous-silicate melts determines *supersolidus* conditions in the mantle-wedge peridotite (thick red line FB^48,49^) at depths as shallow as ~180 km. Keys to mineral breakdown and *solidus* curves. Continuous lines: in brown, wet *solidus* and second critical end-point of pelite (S04)^46^; in grey, carbonated pelite *solidi* with bulk H_2_O and CO_2_ contents in wt% (GS)^45^; in green, part of the *solidus* of carbonated basaltic eclogite (D04)^47^ and hydrated and carbonated gabbro (P15)^50^; in red, *solidi* of dry peridotite (H00)^51^, water saturated peridotite (G14)^52^, dry carbonated peridotite (DH)^53^, and potassium-rich hydrated carbonated peridotite (FB, as compiled by ref.^54^: 0.40–0.63 wt% H_2_O and 1.99–3.21 wt% CO_2_ after ref.^48^; 2.22 wt% H_2_O and 3.63 wt% CO_2_ after ref.^49^, the dashed part is inferred). Dashed lines: parts of amphibole-, zoisite-, lawsonite- and phengite-out curves in basalts (SP)^55^ and pelites/greywakes (PS)^56^; part of the lawsonite-out curve in a CASH system (P94)^57^; part of antigorite-, talc- and chlorite-out curves in ultramafic system (UT^58^, P03^59^, BG^60^). Image generated using Inkscape v0.91 (https://inkscape.org).

Here, we use a cellular tomographic model based on seismic surface waves (see Methods and Fig. 1a) to explore the shear wave velocity (Vs) structure of the supra-slab Adriatic mantle in much greater detail as compared with previous work^6,11,12^. Surface wave tomography, unlike body wave tomography, does not require an *a priori* reference model^13–15^. Our model provides a reliable estimate of the Vs layered structure and its uncertainties to ~350 km depth^14,16,17^. The slab structure provided by body wave tomography^6^, since its uncertainties are not easily quantifiable^15^, can be used as ancillary information. As shown in Fig. 1c, our tomographic model highlights a sharp velocity drop at ~100 km depth that is particularly evident in cross section A-A’. We interpret this drop to mark the boundary between the Adriatic lithosphere and the underlying asthenospheric mantle. The thickness of the Adriatic lithosphere is similar to the thickness of the European lithosphere, as independently constrained by array analysis of seismic surface waves^18^. An additional strong velocity drop is observed in the Adriatic asthenospheric mantle at ~180 km depth (Fig. 1c). This low velocity anomaly (Vs = 4.0–4.2 km/s) is recognised over an area of ~10^5^ km^2^ (light blue cells in Fig. 1a), and is exclusively found within the mantle sandwiched between the European and the Adriatic slabs (yellow and purple colours, respectively, in Fig. 1c). The spatial relationships between this anomaly and the nearby slabs imply that the anomaly formed when the slab structure was already fixed. Recent palinspastic reconstructions^8^ and available plate motion constraints (grey arrows in Fig. 1b) indicate that the slab configuration observed today was acquired during the Neogene (see cartoons in Fig. 2). This low-velocity anomaly is thus a recent feature of the Adriatic upper mantle. It could be the result of melts released in the Neogene from the stagnant European slab, during progressive thermal reequilibration with the adjacent mantle. No mantle plume cutting across the European slab is documented by recent body wave tomographic models^6,12^, ruling out the potential role of deeper mantle processes in determining the observed reduction in shear wave velocities.

## Carbon evolution during Alpine subduction

Carbon release to the mantle wedge strongly depends on the composition and metamorphic evolution of the subducting lithosphere. Based on the composition of the oceanic slivers originally belonging to the Alpine Tethys, and now accreted in the Alpine belt, various proportions of serpentinised mantle, basalts and sedimentary rocks - such as cherts, limestones, greywackes and (calcareous) pelites - were subducted at the Alpine trench since the Cretaceous^7,8^. Direct evidence of the metamorphic evolution of these rocks during oceanic subduction (step 1 in Fig. 2) is provided by the prograde P-T paths of exhumed oceanic (U)HP rocks such as the Viso (VI in Figs 1, 2)^19^ and Zermatt-Saas (ZS in Figs 1, 2)^20^ metaophiolites. The coesite-bearing ophiolitic slice of Cignana, in the Zermatt-Saas unit, was subducted to ~110 km depth ~45 Myr ago. These rocks experienced breakdown of amphibole (Amp) and zoisite (Zo) in metabasics^20^, and likely breakdown of zoisite in metasediments, but dehydration of antigorite (Atg) in ultrabasic rocks was largely incomplete. Dehydration was also incomplete during subsequent continental subduction (step 2 in Fig. 2), as documented by the coesite-bearing continental slice of Brossasco-Isasca, in the Dora-Maira unit (DM in Figs 1 and 2)^21^, which reached depths of ~140 km ~35 Myr ago. The Brossasco-Isasca rocks experienced breakdown of zoisite in metapelites, of zoisite and antigorite in impure marbles, and of chlorite (Chl), talc (Tlc) and phlogopite in Mg-metasomatic rocks^21,22^, but the low paleogeothermal gradient during subduction prevented the breakdown of phengite (Ph).

Carbonates can escape breakdown during cold subduction^23^. In the Alpine subduction zone, dissolution of part of the carbonates was favoured by aqueous solutions released at (U)HP conditions^24^. However, limited metamorphic carbonate destabilisation in continental rocks^22^ suggests that major amounts of subducted carbonates survived decarbonation (CO_2_-rich fluids or melts release) and were probably dragged beyond 120–140 km depths by the downgoing slab.

According to geophysical evidence and paleotectonic reconstructions^6,8^, over 200 km of Tethyan oceanic lithosphere was consumed at the Alpine trench since the Cretaceous, but only a limited amount of this lithosphere was accreted in the Alpine belt and escaped deep subduction. The metamorphic evolution of these rocks at asthenospheric depths, in the lack of direct petrologic evidence, can be inferred by considering the computational thermal models formulated for modern subduction zones^25^. Predicted slab geothermal gradients in the uppermost mantle are largely consistent with those recorded by exhumed Alpine rocks at the same depth range. The yellow arrows A and B in Fig. 2 indicate the temperature-depth trajectories modelled for the Lesser Antilles subduction zone (case D80 in ref.^25^). They show temperatures at the top of the slab that are similar to those recorded by the Brossasco-Isasca slice. Along these cold subduction paths, the breakdown of lawsonite (Lws) in metabasics is predicted at depths larger than 100 km, and dehydration of ultramafic rocks is possibly completed at depths no greater than 180 km. Substantial amounts of aqueous fluids, potentially able to destabilise carbonates, are thus released at sub-arc depths^21,26^. However, carbonates and hydrous minerals (i.e., phengite) are stable both in metasediments and metabasics along the considered subduction paths, which run within the subsolidus field parallel to the main *solidus* slopes of carbonated and/or hydrous crustal rocks to depth of 200–250 km (Fig. 2).

## Carbon sequestration in the Alpine mantle wedge

Since the late Oligocene, the stagnant European slab underwent progressive thermal reequilibration towards ambient mantle conditions^27^ (thick black line in Fig. 2). The expected temperature increase at the slab interface (yellow arrow C in Fig. 2) promoted further dehydration of metabasics at 220–250 km depth, via breakdown of lawsonite (at ~800 °C) and phengite (at ~1000 °C), and finally induced breakdown of carbonates in both calcareous metasediments (at ~1000 °C) and carbonated metabasics (at ~1200 °C) (step 3 in Fig. 2).

**Figure 3:**
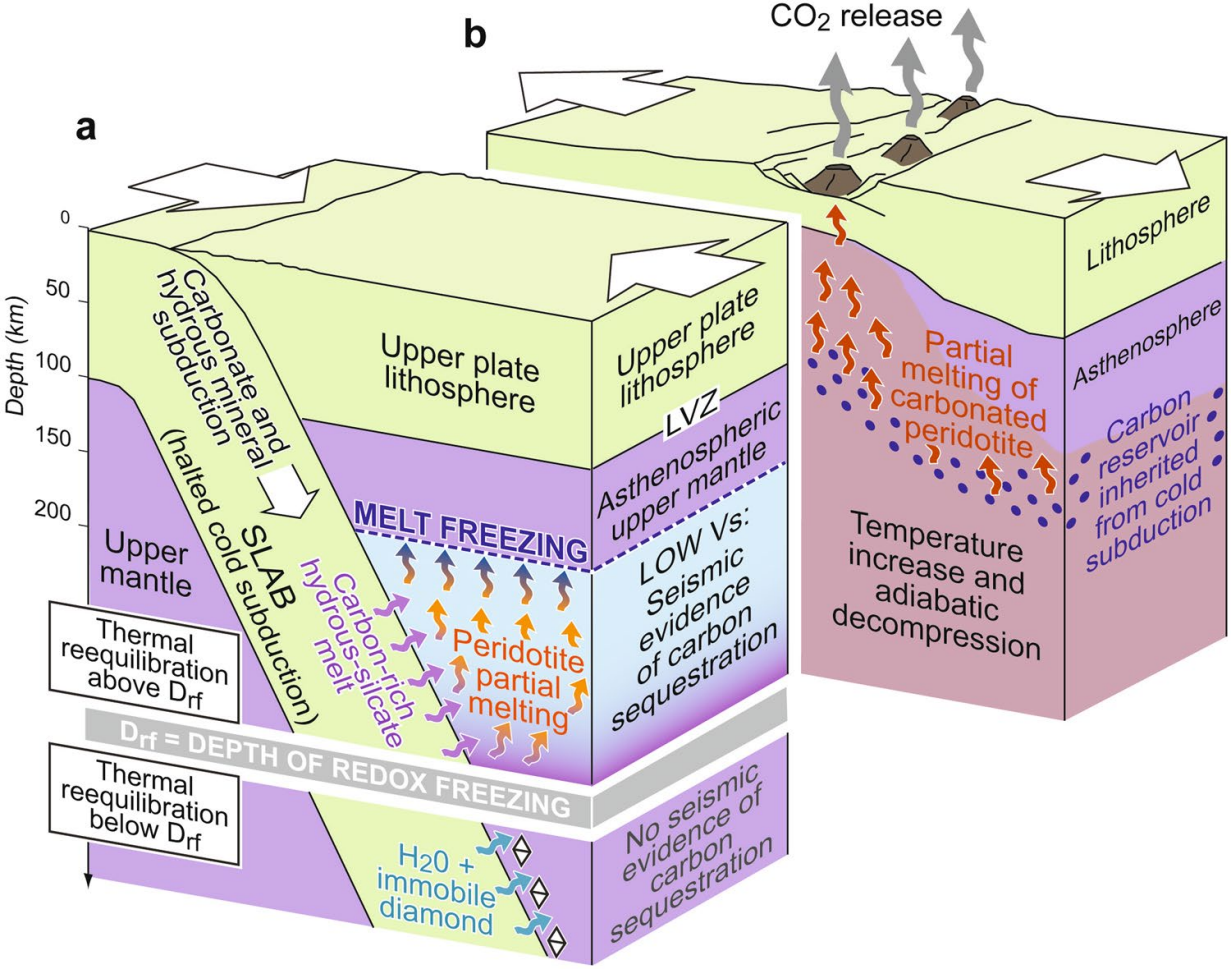
Seismic evidence of carbon sequestration in the upper mantle by cold subduction and delayed CO_2_ release. (**a**) Breakdown of carbonates and hydrous minerals during thermal reequilibration of a cold stagnant slab generates carbon-rich hydrous-silicate melts, that infiltrate the overlying mantle wedge inducing partial melting of the mantle peridotite. The resulting network of carbonate-silicate melt reduces the seismic shear wave velocity (Vs) at depths as shallow as ~180 km, where this carbon-rich melt is solidified. The low Vs layer in the supra-slab asthenosphere thus provides direct evidence of long-term carbon capture and storage in the upper mantle. However, when breakdown of carbonates and hydrous minerals occurs below the depth of redox freezing, carbon sequestration has no seismic evidence because carbon is immediately converted to diamond, and released fluids cannot activate partial melting. (**b**) Carbon stored in the asthenospheric mantle during cold subduction is remobilised at a later stage of the plate tectonic evolution, leading to rapid CO_2_ outgassing with potential harmful effects for the biosphere. Image generated using Inkscape v0.91 (https://inkscape.org).

In the asthenospheric mantle, subducted oxidised carbon can be present as crystalline carbonate or as carbonate-rich melts^28^. Owing to the slope of the carbonated hydrous peridotite solidus (thick red line in Fig. 2), carbon-rich supercritical fluids generated along the interface of the stagnant European slab could have induced melting in the overlying mantle wedge. Low density and low viscosity allow efficient extraction and rising of carbonate-rich melts in the Adriatic asthenosphere. We interpret the drop in seismic shear wave velocities observed at depths larger than 180 km (Fig. 3a) as an effect of the resulting melt network. If melt completely wets grain-boundaries, as demonstrated by laboratory studies for the forsterite +H_2_O system at P > 7 GPa (ref.^29^), even a small amount of melt (e.g., 1%) can produce remarkable velocity drops of 20–30% (ref.^30^).

The key observation of Fig. 2 is that the mantle geotherm crosses the carbonated hydrous peridotite solidus at about 5.6 GPa and 1100 °C. Therefore, mobile carbonate-melt stability is limited to a depth of ~180 km. Above this depth, attainment of peridotite subsolidus conditions requires that carbon is sequestered as magnesite^28^, escaping immediate release by magmatic activity (Fig. 3a). The seismic velocities in the resulting mantle do not show variations, in agreement with the geophysical record (Fig. 1).

A different process of carbon sequestration would be expected if the cold stagnant slab was reequilibrated at greater depths. In a mantle where oxygen fugacity is controlled by Fe^2+^/Fe^3+^ exchange, metal saturation is predicted at depths larger than 200–250 km^3^ ^31^ (Fig. 3a). Since carbonates are not stable at these redox conditions, carbon would be immediately reduced to immobile diamond^3^, and would be sequestered in the mantle. We predict that at depths greater than 200–250 km, there would be no apparent drop in shear wave velocity.

## Implications for carbon budgets and long-term climate trends

The petrological and geodynamical interpretation of the velocity structure of the Alpine mantle wedge suggests that carbon sequestration is active. The Alpine case is particularly favourable for the detection of long-term carbon storage by seismic methods, but the same process may take place without any clear-cut geophysical evidence in other cold subduction zones, for example around the Pacific^4^. This suggests that our model carbon evolution (Fig. 3a) may well have general validity.

Freezing of carbonate-rich melts above ~180 km under the Alps implies that carbon is effectively sequestered in the upper mantle without immediate release. Subducted carbon is possibly remobilised at a later stage of the plate tectonic evolution, for example by thermal increase or redox melting of carbonated peridotites^3^ or adiabatic decompression during continental breakup at the onset of a new Wilson cycle^5^ (Fig. 3b). These processes may occur hundreds of millions of years after subduction ceases, leading to major heterogeneities in the upper-mantle carbon content, with possible formation of large-scale carbon reservoirs at asthenospheric depths.

It has been recently proposed that massive CO_2_ degassing in active rift systems would provide a viable link between continental fragmentation and long-term climate change^32,33^. In the East African rift system, extrapolation of measurements of diffuse CO_2_ degassing away from volcanic centres^32^ imply a carbon flux (~19 Mt C yr^−1^) that is comparable to the present-day emission budget from the entire mid-ocean ridge system^2,32^. During past episodes of supercontinent breakup, widespread continental rifting may have determined even greater short-term carbon outputs of hundreds of Megatons per year^33,34^.

Peaks of atmospheric CO_2_ concentrations recorded through Earth history during supercontinent breakup events are possibly linked to mass extinctions^35^. If our model of carbon capture and storage by cold subduction is accurate, previous estimates of carbon release during continental rifting^32^ may just provide a lower bounding estimate on Earth’s CO_2_ emissions during future episodes of continental breakup. We can thus predict peaks of CO_2_ outgassing higher than in the past, because carbon sequestration in the upper mantle has been favoured during the Phanerozoic by the dominantly cold nature of subduction zones^4,5^.

Natural CO_2_ emissions are not comparable with present-day anthropogenic contributions from fossil fuels and industry (~10 Gt C yr^−1^)^36^. However, massive and rapid increase in atmospheric natural CO_2_ concentration over geologic timescales, as predicted by our model, may have harmful effects, determining a sharp temperature rise that would adverse the habitability of our planet.

The potential occurrence of recycled carbon within the upper mantle of continental rift zones supports the conclusion that the Earth’s carbon cycle is not limited to subduction zones^1,2,5,33^. This implies that the carbon cycle is imbalanced on the time scale of a single plate-tectonic cycle. Whereas mantle carbon sequestration during cold subduction may partly counterbalance the long-term carbon increase in the lithosphere, oceans, and atmosphere determined by CO_2_ outgassed from ridges and oceanic islands^2^, massive recycled-carbon release from upper-mantle reservoirs is expected during supercontinent breakup.

## Methods

The Vs cellular model with a 0.5° × 0.5° lateral resolution utilised in this work is a refinement of the 1° × 1° cellular model, based on the inversion of dispersion data, presented by Brandmayr *et al*.^14^. The following methods are applied in sequence: (a) frequency-time analysis, (b) surface wave tomography, (c) “hedgehog” non-linear inversion of cellular dispersion curves, and (d) optimisation of the non-linearly inverted models to define the preferred model. Our 0.5° × 0.5° model exploits the database of project EuCrust 07^37^ to define the physical properties (Vs and density) and the thickness of the sedimentary cover, the average seismic velocity of the upper crust, and the depth of the Conrad discontinuity.

### FTAN analysis

The seismic record from national and international seismic networks is analysed by an interactive group velocity – period filtering method (FTAN)^38^, which uses multiple narrow-band Gaussian filters and maps the waveform record in a two-dimensional domain: time (group velocity) – frequency (periods). The measurement of group velocity of Rayleigh and/or Love waves is performed on the envelope of the surface-wave train across a broad period band from fractions to hundreds of seconds. Based on available information on event hypocenters^39^, we considered epicenter-to-station paths shorter than 3000 km to get reliable measurements of group velocity at periods ranging from 5 to 80 s. Published phase-velocity measurements for Rayleigh waves in the 15 to 150 s period range are additionally considered to increase the penetration depth of the considered data set.

### Surface wave tomography

We use the two-dimensional tomography based on the Backus–Gilbert method to determine the local values of the group and/or phase velocities for a set of periods^40^, and map horizontal (at a specific period) and vertical (at a specific grid knot) variations. Local values of group and phase velocities are calculated on a predetermined grid of 1° × 1° for set of periods in the range from 5 s to 80 s for group velocities and from 15 s to 150 s for phase velocities and determine the vertical resolving power of the dataset. The lateral resolution of the tomographic maps is defined as the average size (L) of the equivalent smoothing area and its elongation. The local dispersion curves are assembled at each grid knot from the tomographic maps. The group and/or phase velocity value is included in the dispersion curve if L is below a threshold specific of the period (L < 300 km at 5 s and <600 km at 80 s for group velocities; L < 500 km at 15 s and < 800 km at 80 s for phase velocities) and the stretching of the averaging area of all considered values is <1.6. Each cellular dispersion curve is then calculated as the average of the local curves at the four corners of the cell, and the single point error for each value at a given period is estimated as the average of the measurement error at this period and the standard deviation of the dispersion values at the four corners. The value of group velocity at 80 s is calculated as an average between our tomography results and the results of a global study^41^, and the single point error for group velocity at this period is estimated as the r.m.s. of the errors of our data and those of the global data set. The r.m.s. for the whole dispersion curves (group or phase velocity) are routinely estimated as 60–70% of the average of the single point errors of the specific cellular curve^13^.

### “Hedgehog” non-linear inversion

The cellular dispersion curves compiled from surface wave tomography are inverted by the “hedgehog” non-linear inversion method^13,17^. In the inversion scheme, Vs and thickness are the independent parameters, Vp is dependent on Vs (in general Vp/Vs = 3^1/2^), and density is determined according to the Nafe-Drake relation^42,43^. The group and phase velocities of the Rayleigh waves (fundamental mode) are computed for each tested structural model. To avoid overinterpretation of the inversion results, the details allowed in the structural models are consistent with the resolving power of the inverted data set^17^. The model is accepted if the difference between the computed and measured values at each period are less than the single point error at the relevant period, and if the r.m.s. values for the whole group and phase velocity curves are less than the given limits.

### Optimisation

All the solutions for each cell are simultaneously processed with an optimised smoothing method to select the representative solution that minimises the local lateral velocity gradient^16^. Three optimisation algorithms (Local Smoothness Optimisation, Global Smoothness Optimisation and Global Flatness Optimisation) are applied hierarchically to search for the minimising solution within five neighbouring cells, along a row of cells, and through the whole study area^14,16^. Solutions for cells bordering the 0.5° × 0.5° study area are fixed according to the 1° × 1° model. Results are appraised using independent dataset concerning Moho depth and heat flow. Vp values from the literature^44^ are used to reduce the uncertainty ranges of Vs in each layer of the final model, by keeping a Vp/Vs ratio in the mantle as close as possible to 1.82.
